# Heterosis and differential gene expression in hybrids and parents in *Bombyx mori* by digital gene expression profiling

**DOI:** 10.1038/srep08750

**Published:** 2015-03-04

**Authors:** Hua Wang, Yan Fang, Lipeng Wang, Wenjuan Zhu, Haipeng Ji, Haiying Wang, Shiqing Xu, Yanghu Sima

**Affiliations:** 1 Department of Applied Biology, School of Biology and Basic Medical Sciences, Medical College of Soochow University, Suzhou 215123, China; 2 National Engineering Laboratory for Modern Silk, Soochow University, Suzhou 215123, China; 3 Department of Cell Biology and Genetics, College of Life Sciences, Nankai University, Tianjin 300071, China; 4 Department of Immunology, Nankai University School of Medicine, Tianjin 300071, China

## Abstract

Heterosis is a concern to all breeders, but the mechanism of heterosis remains unknown. In F_1_ organisms, genetic material is inherited from the two parents and theoretically, heterosis might be caused by differences in gene expression or modification. Differential gene expression was analyzed in hybrids and parents in *Bombyx mori*. The results showed that there were significant changes in gene expression in the fat body involving biological regulation, cellular and metabolic processes. Consistent trends in expression patterns covering different hybrid combinations were seen in 74 genes. Moreover, these differential gene expression patterns included overdominance, dominance, and additive effects. By correlating these patterns with economic traits, a potential relationship was found. Differential gene expression was seen in different cross combinations and in different sexes. In addition, a regulatory mechanism involving metabolism and ErbB signaling pathways was also found, suggesting that such a network might also be related to heterosis in *Bombyx mori*. Together, our data provide a comprehensive overview and useful resource for transcriptional analysis of heterosis of *Bombyx mori*.

Heterosis, or hybrid vigor, was first proposed by Shull in 1914 to describe the phenomenon of a hybrid offspring with enhanced viability and developmental rates compared with its parents^1^. Current hypotheses to explain heterosis include a dominance model proposed by Bruce that emphasizes the relationship between dominant and recessive genes^2^,^3^,^4^. Another is an overdominance model proposed by Shull and East that emphasizes heterozygosity^5^. Yu et al. proposed an epistasis theory that emphasizes nonadditive genetic effects based on the overdominance hypothesis^6^. Recent progress has suggested additional mechanisms which may explain heterosis in *Arabidopsis* and maize, including expression of small RNA (sRNA)^7^ and DNA methylation^8^. To examine these mechanisms, transcriptional or gene expression profiles have been completed in maize^9^, rice^10^,^11^, and pufferfish^12^. In the maize study, all possible modes of gene action were examined in a global comparison of gene expression in a maize F_1_ hybrid and its inbred parents^13^. These analyses have deepened our understanding of heterosis, but none completely and independently explains this complex phenomenon.

Genetic materials of hybrid offspring are inherited from the two parents. Theoretically, no new genes are produced, so heterosis is likely caused by differences in gene expression or qualitative or quantitative modification. Differences will also be affected by environment^14^,^15^. According to genomic and epigenetic principles, offspring exhibit advantages in growth, stress resistance and adaptability because of interactions between alleles of parental genomes that change the regulatory network of related genes. Currently, whole-genome transcriptome analyses of many parental and hybrid offspring plants have been completed, and correlations between differential gene expression patterns and traits have been demonstrated^9^,^14^,^15^,^16^,^17^. Romagnoli et al.^16^ as the first to analyze differences in gene expression in root tips between hybrid and parental maize. The results suggested a correlation between heterosis and differential gene expression. Bao et al.^17^ studied heterosis of the *LYP9* rice strain and its parents using serial analysis of gene expression (SAGE) technology. The results showed that upregulated genes in *LYP9* were mainly involved in leaf photosynthesis, root nitrogen uptake and rapid root growth. Wei et al.^18^ carried out genome-wide microarray analysis of *LYP9* at different growth stages and in different tissues and found that 10.6% of genes were differentially expressed. Five basic differential expression patterns were summarized: parent silence, parent-specific expression, hybrid-specific expression, expression in both hybrids and a single parent, and co-expression.

Although the use of transcriptomics technology has led to important breakthroughs in the study of heterosis in plants, research on heterosis in animals is difficult and requires accounting for the effects of different parental combinations (cross, reciprocal cross) and different sexes on animal heterosis. We chose *Bombyx mori (B. mori)* as a research subject because the entire genome of *B. mori* is sequenced and a lepidopteran model insect as determined by the International Association of Invertebrates^19^, which has promoted *B. mori* as a model organism not only for lepidopterans but also for general biology^20^. *B. mori* is an excellent model to investigate the mechanism of heterosis for the following reasons: (i) They are easily fed with fresh mulberry leaves; (ii) the number of eggs produced by *B.mori* are sufficiently high that a large number of samples can be procured at one time for statistical analysis; (iii) analysis of heterosis covering economic traits of *B.mori* has been well characterized and there are many mature appraisal systems widely utilized in silkworm breeding. Therefore, we used *B. mori* to investigate correlations between gene expression patterns and heterosis.

## Results

### Heterosis of silkworm economic characters

Offspring of crosses and reciprocal crosses were superior to the parents 75xin and “7532” for all traits (Figure S1). The larva-pupa survival rate and pupal unified survival rate of the crosses (75xin female × “7532” male) F_1_ offspring were higher than reciprocal cross F_1_ offspring (“7532” female × 75xin male) (Figure S1A and B), while dead worm cocoon rates of the crosses F_1_ progeny were lower than rates for reciprocal cross F_1_ (Figure S1D). Cocoon shell weights of F_1_ females and males exhibited high heterosis (Figure S1E), while sex differences were seen in the whole cocoon, pupal weight and cocoon shell percentages (Figure S1E–G). Most of the heterosis rates showed that crosses F_1_ offspring had more favorable economic traits than those of the reciprocal cross F_1_ (including the length and diameter of silk fiber (Figure S1I and J)). Moreover, F_1_ females had higher heterosis for cocoon shell, whole cocoon, pupal weights and diameter of silk fiber than males. Heterosis of males for cocoon shell percentage and length of fiber was higher than females (Figure S1K).

### Sequencing quality evaluation and distribution

**Figure 1:**
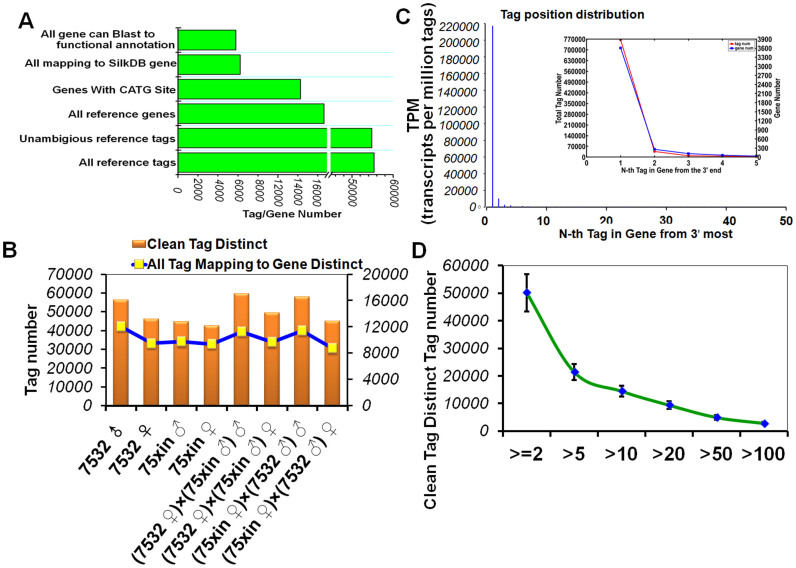
Distributions of tags and genes. (A) Data on reference genes in this study. (B) Distribution of distinct clean tags and genes. (C) Positional distribution of all tags that could be mapped to a gene. Position distribution vector (scalar figure) of the distance of a tag aligned to a reference gene from a 3′-end restriction site. Insert: vector of changes in tag types increased with sequencing number. (D) Distribution of distinct clean tag copy numbers from the eight libraries. The error bars indicate standard deviation.

Clean tags were obtained after removal of impurities (Figure S2), and the final number of clean tags ranged from 42,584 to 59,685 (Figure S3). Our results suggested that most tags had copy numbers greater than 100, while low-expression tags (with copy numbers less than 5) were abundant in tag types (Figure S3A–H), consistent with the heterogeneity and redundancy of mRNA expression^21^. When the sequencing number reached 2.5 million, all gene numbers were saturated (Figure S4). In our libraries, the number of all reference genes was 14,623 and the number of genes with a CATG site was 12,264. The number of genes that could be mapped to SilkDB (http://silkworm.genomics.org.cn/) was 6227 (Figure 1A). Between 42,584 and 59,658 distinct clean tag types were detected in our libraries (Figure 1B and Figure S5). The percentage of genes located on the first CATG site from the 3′-end was 99% (Figure 1C). The copy number of most genes indicated their low abundance with only a small number of genes showing highly abundant expression (Figure 1D).

### Venn analysis of all genes in F_1_ and parent libraries

**Figure 2:**
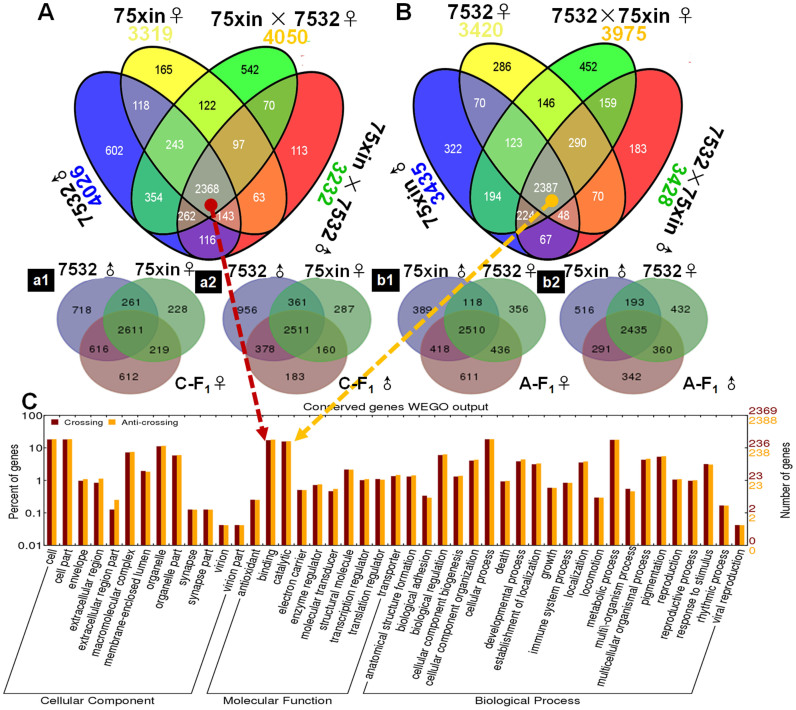
Venn diagram and GO analysis of shared genes. (A) and (B) Venn diagram of unique and shared genes among the eight libraries. “a1”, “a2”, “b1”, “b1” displayed the detail venn diagram between F_1_ and parents. (C) GO analysis of all shared conserved genes by WEGO (http://wego.genomics.org.cn/cgi-bin/wego/index.pl).

**Figure 3:**
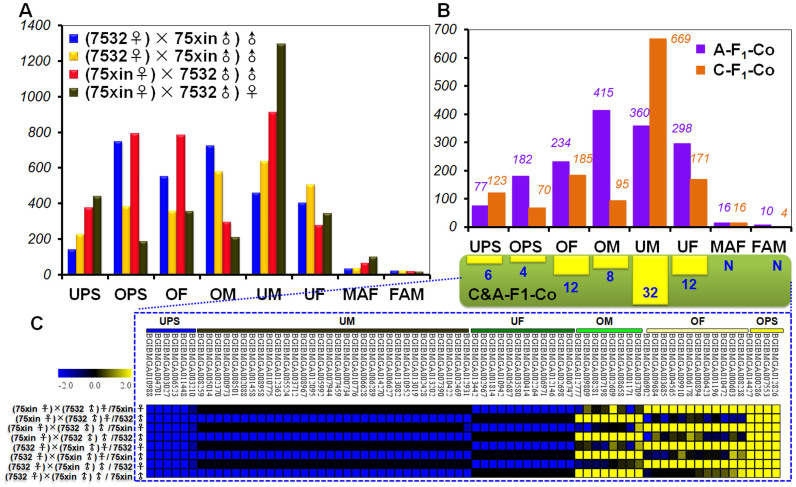
Gene expression patterns clustered by hybrid combination. (A) Distribution of eight patterns by gender and combination. The eight differential expression patterns were No Difference(ND), Over Parents(OPS), Under Parents(UPS), Under Female(UF), Under Male(UM), Over Female(OF), Over Male(OM), Between Female and Male(FAM), Between Male and Female(MAF). (B) Trends in gene expression patterns. The differential expression pattern of F_1_ males and F_1_ females with the greatest number was consistently OM, while the most common pattern for crosses was UM. In both of the two hybrids, the patterns with the lowest numbers were MAF and FAM. “A-F_1_-Co” indicated the co-expression genes in reciprocal cross group, “C-F_1_-Co” indicated the co-expression genes in cross group, “C&A-F_1_-Co” indicated the co-expression genes in cross and reciprocal cross group. (C) Clustering figure for 74 consistently trending genes. All the vertical axis shows the number of genes.

Venn diagrams were plotted for eight libraries before variance analysis. A unique gene specifically expressed in only one library exhibited the greatest difference between the male parents and the F_1_ females (Figure 2A and B). For 75xin females × “7532” males (cross), the number of unique genes was 602 for 75xin females, 165 for “7532” males, 113 for F_1_ males, and 542 for F_1_ females (Figure 3A). For “7532” × 75xin (reciprocal cross), the number was 286, 322, 183 and 452 (Figure 2B). The number of genes that could be detected in both F_1_ offspring and parents was 2368 for the cross combination and 2387 for the reciprocal cross. Among relatively conserved genes, expression of about 80 ribosomal protein genes was the most abundant and stable. Gene ontology analysis (GO) of shared genes indicated that distributions of the type and quantity of these genes were similar in basic function classifications such as cellular component, molecular function, and biological process, which most genes involved in macromolecular complexes, binding, or metabolic processes (Figure 2C).

### Pattern classification and distribution of differentially expressed genes

Differentially expressed genes were classified by patterns (Table S1). Copy numbers for tags corresponding to each gene were converted to transcript per million clean tags (TPM) as relative gene expression levels for the libraries. Fold differences were the binary logarithms of the TPM ratios of F_1_ offspring to parents. Fold changes ≤−2 or ≥2 were considered significant. Using fold differences, basic differential gene expression patterns between F_1_ offspring and their parents were classified into 9 types (Table S1).

The eight differential expression patterns indicated the existence of differences in pattern distributions between males and females and between different hybrid combinations. In cross combinations, the number of differentially expressed genes between F_1_ males and parents in the ND group was 1746, with most differentially expressed genes in UM (911), OPS (792), and OF (784). In F_1_ females, the number of differentially expressed genes in UM was the largest at 1295, followed by UPS at 439, and OF at 356 (Figure 3A). For reciprocal crosses, OPS and OM were the most common (747 and 722, respectively) differential expression patterns between F_1_ males and parents, followed by 142 for UPS. Whereas the most common expression patterns between F_1_ females and parents were UM, OM, OPS and UPS (634, 576, 381, and 224, respectively), the total for MAF and FAM was 57 (Figure 3A). In reciprocal crosses, the differential expression pattern of F_1_ males and F_1_ females with the greatest number was consistently OM, while the most common pattern for crosses was UM. In both of the two hybrids, the patterns with the lowest numbers were MAF and FAM (Figure 3B). Only 74 differentially expressed genes showed a consistent trend in expression patterns between cross F_1_s and reciprocal cross F_1_s. Among these genes, 32 genes belonged to UM, 8 to OM, 12 to UF, 12 to OF, 4 to OPS, and 6 to UPS (Figure 3C).

### GO and KEGG analysis of differentially expressed genes

**Figure 4:**
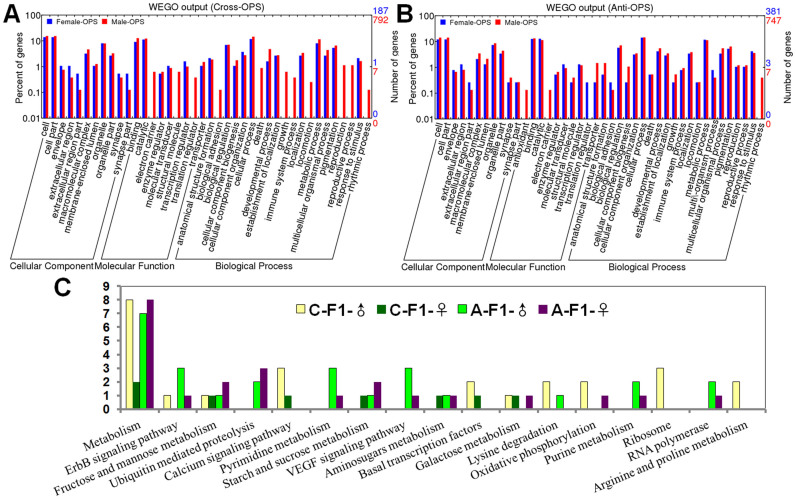
GO and KEGG pathway enrichment analysis of OPS genes in different combinations. (A) GO analysis of differentially expressed genes between cross and F_1_. (B) GO analysis of differentially expressed genes between reciprocal crosses and F_1_. (C) KEGG analysis of differentially expressed genes between cross and reciprocal crosses.“C-F_1_-♂” indicated the co-expression genes in cross F_1_ males, “C-F_1_-♀” mean cross F_1_ females.“A-F_1_-♂” indicated the co-expression genes in reciprocal cross F_1_ males, “A-F_1_-♀” mean reciprocal cross F_1_ females.

GO and KEGG (Kyoto Encyclopedia of Genes and Genomes) analysis of differentially expressed genes in the OPS pattern was performed and both similarities and differences between cross and reciprocal crosses were noted, as well as differences between sexes in the same combination (Figure 4). GO analysis of different hybrid combinations and sexes showed that most genes were in cellular components including cell, cell part, macromolecular complex, organelle, and organelle part; molecular functions including binding and catalytic; and biological processes including biological regulation, cellular process, metabolic process, and pigmentation. For cross combinations, unique differentially expressed genes of F_1_ males not seen in F_1_ females were in categories of electron carrier, structure molecule, translation regulator, biological adhesion, death, growth, immune system process, locomotion, reproduction, reproductive process, and rhythmic process (Figure 4A). For reciprocal crosses, unique genes of F_1_ males were in three areas: antioxidant, electron carrier, and rhythmic process (Figure 4B). KEGG analysis showed the most genes in metabolism pathways with 8 for cross F_1_ males, 2 for cross F_1_ females, 7 for reciprocal cross F_1_ males, and 8 for reciprocal cross F_1_ females. Genes were mainly in metabolism of fructose and mannose, pyrimidine metabolism, starch and sucrose, and amino sugars; they were also in the pathways of ErbB signaling, ubiquitin-mediated proteolysis, calcium signaling, and VEGF signaling (Figure 4C).

### qPCR validation of differentially expressed genes

**Figure 5:**
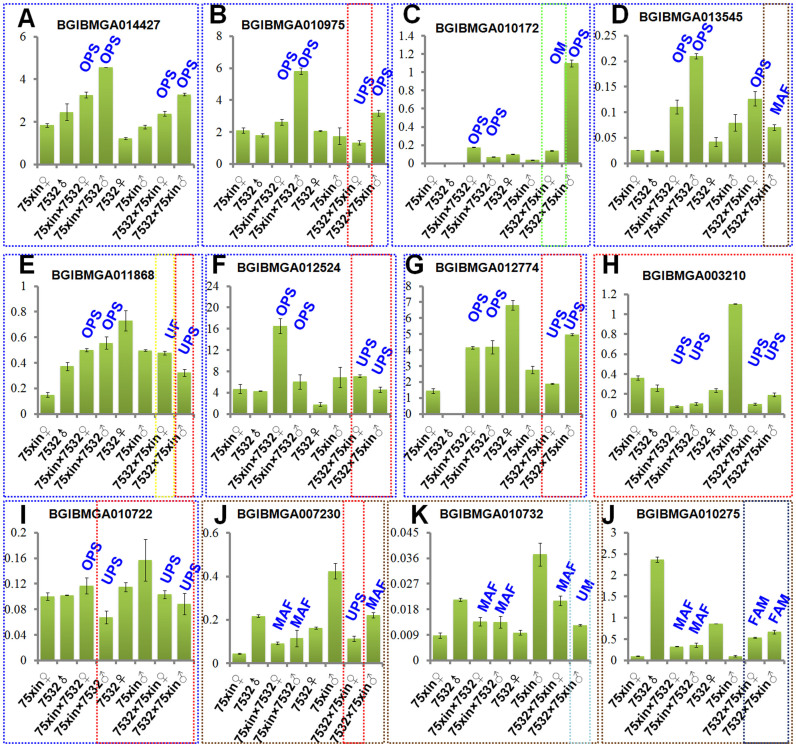
Validation of 12 *B. mori* gene expression levels using qPCR. Known genes were (A) BGIBMGA010975 (*B. mori* homologous FRG1 protein). (B) BGIBMGA010172 (*B. mori* transcription initiation factor subunit 12). (C) BGIBMGA014427 (serine protease precursor). (D) BGIBMGA007230 (*B. mori* elongation factor 1A2). (E) BGIBMGA003210 (*B. mori* Yokozuna transposon). (F) BGIBMGA010722 (*B. mori* facilitated trehalose transporter-like protein Tret1). (G) BGIBMGA010275. (H) BGIBMGA012774. (I) BGIBMGA011868. (J) BGIBMGA012524. (K) BGIBMGA013545. (L) BGIBMGA010732. The error bars indicate standard deviation. All the vertical axis shows the Relative expression of genes.

Six known and six unknown genes with consistent expression trends in F_1_ offspring from cross combinations were selected for validation by quantitative real-time polymerase chain reaction (qPCR) (Figure 5). Except for BGIBMGA010722, gene expression trends were consistent with DGE library results. BGIBMGA010975, BGIBMGA012524, BGIBMGA013545, BGIBMGA010172, BGIBMGA014427, BGIBMGA012774, BGIBMGA011868 in cross F_1_ offspring showed the OPS expression pattern. For reciprocal crosses, the 12 genes analyzed by qPCR showed that in contrast to cross F_1_ offspring, expression of BGIBMGA012774 and BGIBMGA012524 in reciprocal cross F1s had the UPS pattern. For the two combinations, expression patterns were the same for BGIBMGA014427, which showed the OPS pattern, and BGIBMGA003210, which showed the UPS pattern.

### Correlation analysis of gene expression patterns and heterosis

Highly positive correlations were seen between pupal weight and UF (correlation coefficient 0.88, P = 2E-04) and negative correlation between pupal weight and OF (−0.900, P = 0.000129) and OPS (−0.691, P = 2.42E-05). Low positive correlation was seen between pupae weight and OM (−0.537, P = 2.27E-05), UM (−0.356, P = 7.52E-06), UPS (−0.592, P = 0.00017), MAF (−0.670, P = 0.01097). Positive correlation was seen between cocoon shell weight and FAM (0.967), P = 3.38E-05). Whole cocoon weight correlated with UF (0.897, P = 0.0265), FAM (0.693, P = 6.65E-07), OF (−0.887, P = 0.033). Correlation coefficients between cocoon shell percentage OF and OPS were 0.952 (P = 3.92E-05) and 0.924 (P = 7.29E-06), respectively; and between cocoon shell percentage and UF and UM were −0.622 (P = 4.94E-05) and −0.612 (P = 2.27E-06), respectively. A high positive correlation was observed between silk fiber diameter and OM, while the relationship between fiber diameter and UPS was strongly negative (Table 1). These results suggested correlation between differential gene expression patterns and heterosis in *B. mori* that was not fixed and a single pattern.

## Discussion

The results of this study indicated differences in gene expression between different cross combinations and between sexes from the same combination. The gene expression patterns that showed the largest differences for cross females, reciprocal cross females and males compared with parents was UM (13.87%, 6.78% and 17.30%, respectively). These differential gene expression patterns were consistent with the dominance hypothesis, which suggests that a harmful recessive allele from a parent would be inhibited by an advantageous dominant allele from another parent. Thus, the offspring have traits similar to the parent with the dominant allele^18^,^22^,^23^,^24^.

Gao et al. compared the transcriptome of an artificially bred strain of pufferfish, Jiyan-1, with fast growth, good flavor and strong resistance to adverse environments, with its parents *Takifugu flavidus* (female) and *T. rubripes* (male). The OPS expression pattern accounted for 68.7% of all differentially expressed genes^12^. In our experiments, the OPS expression patterns seen in cross females and males, and in reciprocal cross females and males were 3.87%, 15.04%, 8.33% and 15.44%, respectively. The OPS genes were consistent with the overdominance hypothesis, which suggests that heterozygosity is superior to homozygosity in offspring. Heterozygosity usually increases gene expression^13^. Males were a greater proportion of the OPS category than females. These data demonstrated overdominance in heterosis. However, the UPS patterns observed in cross females and males, and in reciprocal cross females and males were 7.16%, 9.08%, 2.94% and 4.90%, respectively, suggesting that gene expression levels in offspring were significantly lower than in parents. Therefore, a low-dominant, or underdominant mode of heterosis also existed in *B. mori*. The coexistence of overdominance and underdominance might be related to microRNA (miRNA) involvement in gene splicing and silencing^25^. For cross F_1_s and reciprocal cross F_1_s, the lowest percentage of all patterns were MAF and FAM, which satisfied the additive effect. The overall results indicated that dominance or overdominance might play a major role in heterosis and account for a dominant position in *B. mori* heterosis, while low dominance and additive effects also existed. This finding is consistent with a previous hypothesis of multiple heterosis mechanisms^13^,^26^,^27^.

GO and KEGG analysis of the differential expression pattern OPS between F_1_ and parents was consistent with previous studies in other organisms^12^,^28^. A number of genes were involved in cellular process, metabolic process, binding, catalytic activity, cell, and organelles, indicating that these functions may be associated with metabolism and accelerated growth and development of F_1_ progeny^28^,^29^. Upregulation of many genes involved in metabolism is consistent with studies of heterosis in *Arabidopsis* *thaliana*^30^. Gao et al.^12^ compared the transcriptome of the artificially bred pufferfish Jiyan-1 with its parents. A total of 2024 differentially expressed genes were clustered to metabolism, nucleic acid binding and catalytic activity, indicating that the biological function of metabolic activity is the most active in heterosis. This differentially expressed gene enrichment of 35 KEGG pathways, with genes involved in metabolism, ion-binding, and kinase activity suggests candidate genes for heterosis and multiple gene regulation in heterosis. In this study, many KEGG pathways were also enriched, many in metabolism-related pathways. This finding has been validated in other species, for example, the thiamine and pyrimidine pathways of nutritional mutants of *A. thaliana* have strong complementary effects^31^. In 2008, Ni et al. reported that epigenetic modifications of circadian clock genes altered expression of downstream genes and pathways in *Arabidopsis*^32^. They suggested that hybrids and allopolyploids gain advantages from the control of circadian-mediated physiological and metabolic pathways, leading to growth vigour and increased biomass. We also investigated known circadian rhythm genes including period (per, BGIBMGA000487), timeless (tim, BGIBMGA006227), Clock (Clk, BGIBMGA000498), cycle (cyc, BGIBMGA003870), vrille (vri, BGIBMGA013421), double-time (dbt, BGIBMGA007304), and shaggy (sgg, BGIBMGA008336), but observed no significant differences in expression between F_1_s and parents, which may be due to height differences between the species.

Currently, however, the mechanism of the effects of many signaling pathways on heterosis remains unclear. Although some differentially expressed genes with significant expression differences have been found, no direct evidence indicates if the upregulation or downregulation of these genes is the only decisive factor in heterosis. Heterosis might be influenced by the results of the interaction of genes in signaling pathways^12^. Our results provide references for hypotheses about correlations between potential pathways and heterosis.

The metabolism of carbohydrates, amino acids and triglycerides is critical for organisms to maintain normal physiological function. Of the differentially expressed genes with an OPS pattern in cross males, involvement was seen in amino acid metabolic and fat metabolic pathways. Two were involved in the TCA cycle. Results on heterosis in maize and barley showed that increased TCA cycle enzyme activity in offspring is related to a high respiratory rate and mitochondrial integrity^33^,^34^. Correlation between heterosis and changes in metabolic function, especially regulation of the TCA cycle and its intermediates has also been shown in *A. thaliana*^35^. Significant differences are seen in fatty acid and myelin metabolism between hybrid cattle, hybrid rice and parents^36^,^37^. Heterosis in *B. mori* is due to accumulation of multiple functions. Results on hybrid rice^38^ show that genes in carbohydrate metabolism, lipid metabolism, energy metabolism and protein degradation are downregulated and genes in amino acid metabolism are either upregulated or downregulated. Our results suggested that genes in these pathways were also either upregulated or downregulated.

Correlation analysis between gene expression patterns and heterosis showed a complex correlation between differential gene expression patterns and multiple silkworm economic traits. These results indicated that heterosis in silkworms was due to overall differences in gene expression and large differences in genes involved in economic traits. These differences need to be elucidated further in followup research.

In summary, transcriptome analysis showed that overdominance was the major mode of gene action in *B. mori* heterosis. A variety of multigene regulation modes including overdominance, dominance, low dominance and additive effects co-existed. These findings suggested that heterosis formation in animals involves multiple molecular regulation mechanisms.

## Methods

### Biological materials

Two parents, 75xin and “7532”, and their cross and reciprocal cross F_1_ hybrids (cross 75xin female × “7532” male and reciprocal cross “7532” female × 75xin male) of *B. mori* were used as experimental materials. Parent 75xin is a Chinese variety and “7532” is a Japanese variety. F_1_ hybrids of 75xin female × “7532” male had excellent disease resistance and resistance to high temperature, high cocoon shell percentage and silk indexes, and high-grade silk. Larva-pupa survival rates, pupa unified vital rates, dead worm cocoon rates and egg quantities of single moths were examined in triplicate. Cocoon shell weight, whole cocoon weight, cocoon shell percentage , pupal weight and diameter of silk fiber of males and females were measured.

### RNA extraction

Total RNA was extracted from fat bodies from larvae three days into the fifth instar stage and using RNAiso Plus (TaKaRa). RNA samples were immediately stored in liquid nitrogen for DGE library construction or qPCR.

### DGE library preparation

The main reagents and supplies were Illumina Gene Expression Sample Prep Kits and Solexa Sequencing Chips (flowcells), and the main instruments were an Illumina Cluster Station and Illumina Genome Analyzer System. All protocols for preparation of DGE libraries were according to the manufacturer's instructions and Gao et al.^12^.

### Bioinformatics analysis

Sequence components with low quality or impurities in the original data were removed. All CATG sites were retrieved from SilkDB (http://silkworm.genomics.org.cn/) and *B. mori* tag libraries containing references were generated. SeqMap was used to match genes, and Blast2go was used to annotate functions of matched genes. Clean tags corresponding to each gene were counted with standardized gene expression level = clean tag number corresponding to each gene number of total clean labels in the sample × 1,000,000^39^.

### GO and pathway analysis

GO analysis was carried out on differentially expressed genes screened using MAS3.0. (http://bioinfo.capitalbio.com/mas3/) Differentially expressed genes were mapped to terms and the number of differentially expressed genes per term was calculated. Pathways with genes were clustered using KEGG and major pathways with differentially expressed genes were obtained.

### Differential gene expression and correlation analysis

Correlation between differential gene expression patterns and heterosis in *B. mori* was analyzed using SPSS 17.0 software. Pearson correlations were determined with the proportion of each pattern in the combination set as an independent variable (F-test). Heterosis of traits (H%) was set as the dependent variable.

### Quantitative polymerase chain reaction

SYBR Premix Ex Taq (Perfect Real Time; TaKaRa) kit were used for qPCR (Table S2), in accordance with the manufacturer's instructions. Reaction volumes were 20 µL and cycling conditions were: 1 minute 95°C and 45 cycles of 95°C for 5 s, 55°C for 10 s and 72°C for 10 s. *BmRP49* was the internal reference.

### Statistical analyses

We used SPSS 17.0 to analyze correlations between the proportion of the eight differential expression patterns and combinations and silkworm traits of pupal weight, cocoon shell weight, whole cocoon weight, and cocoon shell percentage.

## Author Contributions

H.W. performed the main experiments; L.P.W. and Y.F. performed the qPCR experiments; W.J.Z., H.Y.W., H.P.J. helped H.W. performed bioinformatics analysis; Y.H.S.M. and S.Q.X. designed the experiments; and Y.H.S.M. and H.W. wrote the manuscript. All authors read and approved the final manuscript.

## Supplementary Material

Supplementary Information All supplementary information

## Figures and Tables

**Table 1:** The correlation of gene expression patterns with economic characteristics

Pearson Correlation	UM	OM	UF	OPS	OF	UPS	MAF	FAM
Pupae weight	0.243	0.291	0.880	−0.691	−0.900	0.120	0.091	0.666
Cocoon shell weight	−0.537	0.809	0.920	0.063	−0.356	−0.592	−0.670	0.967*
Whole cocoon weight	0.206	0.324	0.897	−0.663	−0.887	0.083	0.053	0.693
Cocoon shell percentage	−0.612	0.085	−0.622	0.924	0.952*	−0.490	−0.486	−0.318
Length of fiber	0.3736	−0.8107	−0.7033	0.0141	0.5157	0.5820	0.3684	−0.7606
Diameter of silk fiber	−0.8034	0.996**	0.6645	0.4409	−0.0960	−0.9140	−0.8132	0.8637

*Correlation is significant at the 0.05 level (2-tailed).

**Correlation is significant at the 0.01 level (2-tailed). All P values for the correlation analysis are shown in Table S3.
