# Supplementary material for: Heterosis and differential gene expression in hybrids and parents in Bombyx mori by digital gene expression profiling
Source: Sci Rep. 2015 Mar 4;5:8750. doi: 10.1038/srep08750 (PMC4348626; doi:10.1038/srep08750)
Supplement: Supplementary Information — All supplementary information [file srep08750-s1.doc]

**Heterosis and differential gene expression in hybrids and parents in *Bombyx mori* by** **digital gene expression profiling**

Hua Wang^1,3, 🞤^, Yan Fang^1,4, 🞤^, Lipeng Wang^1^, Wenjuan Zhu^1^, Haipeng Ji^1^, Haiying Wang^1^, Shiqing Xu^1,2^, **Yanghu Sima**^1,2^,*

^1^ Department of Applied Biology, School of Biology and Basic Medical Sciences, Medical College of Soochow University, Suzhou 215123, China.

^2^ National Engineering Laboratory for Modern Silk, Soochow University, Suzhou 215123, China.

^3^ Department of Cell Biology and Genetics, College of Life Sciences, Nankai University, Tianjin 300071, China.

^4^ Department of Immunology, Nankai University School of Medicine, Tianjin 300071, China.

* Correspondence: **Yanghu Sima**, Department of Applied Biology, School of Biology and Basic Medical Sciences, Medical College of Soochow University, Suzhou 215123, China.

Tel.: +86 0512 65880255; fax: +86 0512 65880255;

**E-mail: simyh@suda.edu.cn**

^🞤^ These authors contributed equally to this work.

**Supplementary Information**


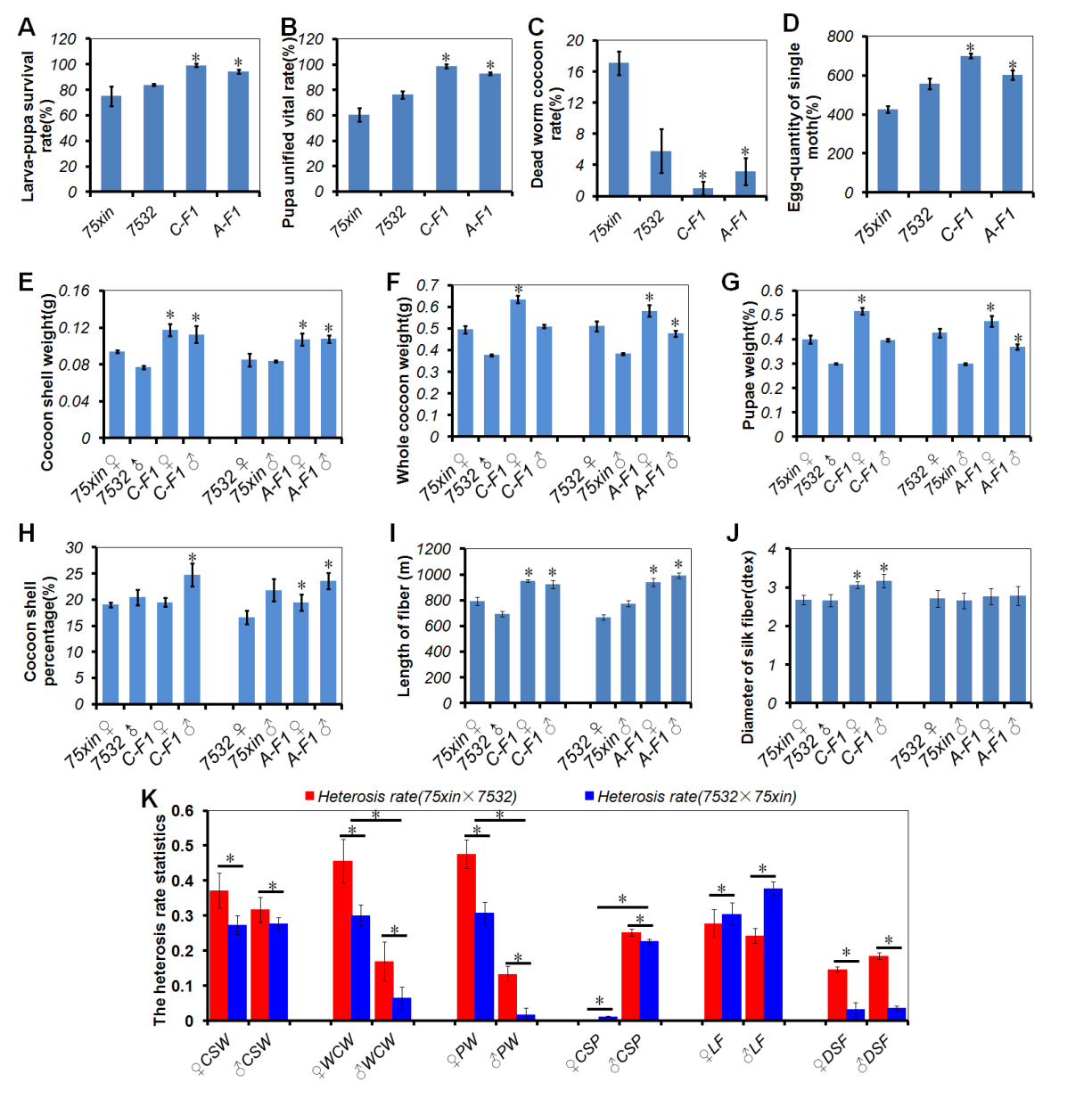


**Figure S1 Heterosis of silkworms for economical characteristics.** (A) Larva-pupa survival rate (%), (B) Pupa unified vital rate (%). (C) Dead worm cocoon rate (%). (D) Egg quantities of single moths (egg grains). (E) Cocoon shell weight (g, CSW). (F) Whole cocoon weight (g, WCW). (G) Pupal weight (g, PW). (H) Cocoon shell percentage (%, CSP).(I) Length of fiber (m, LF). (J) Diameter of silk fiber (dtex, DSF). C-F1 indicates the crosses F1 offpsring and A-F1 means the reciprocal cross F1 offpsring. (K) Heterosis of silkworms. The error bars indicate standard deviation. * indicates p < 0.05 using paired two-tailed T-test.


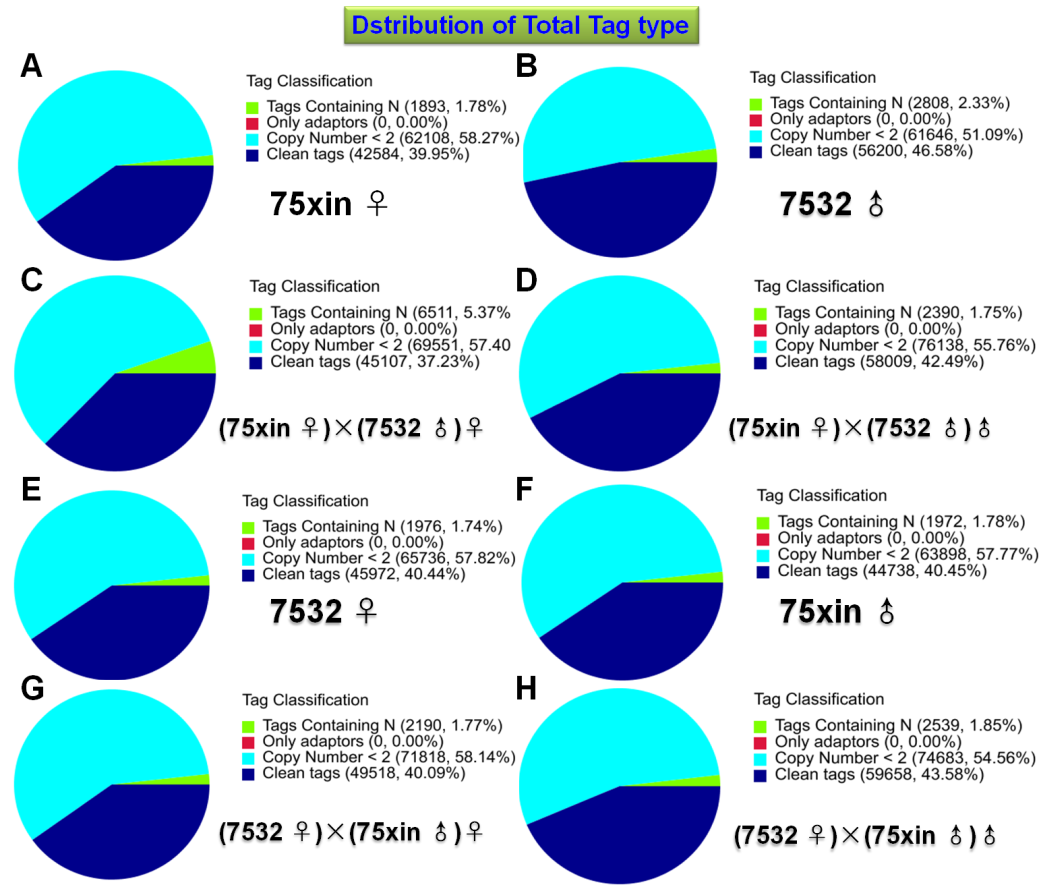


**Figure S2 Distribution of total tag types for eight DGE libraries.** Raw sequences had 3'-adaptor fragments, a few low-quality sequences and several types of impurities. Raw sequences were transformed into clean tags after data processing.


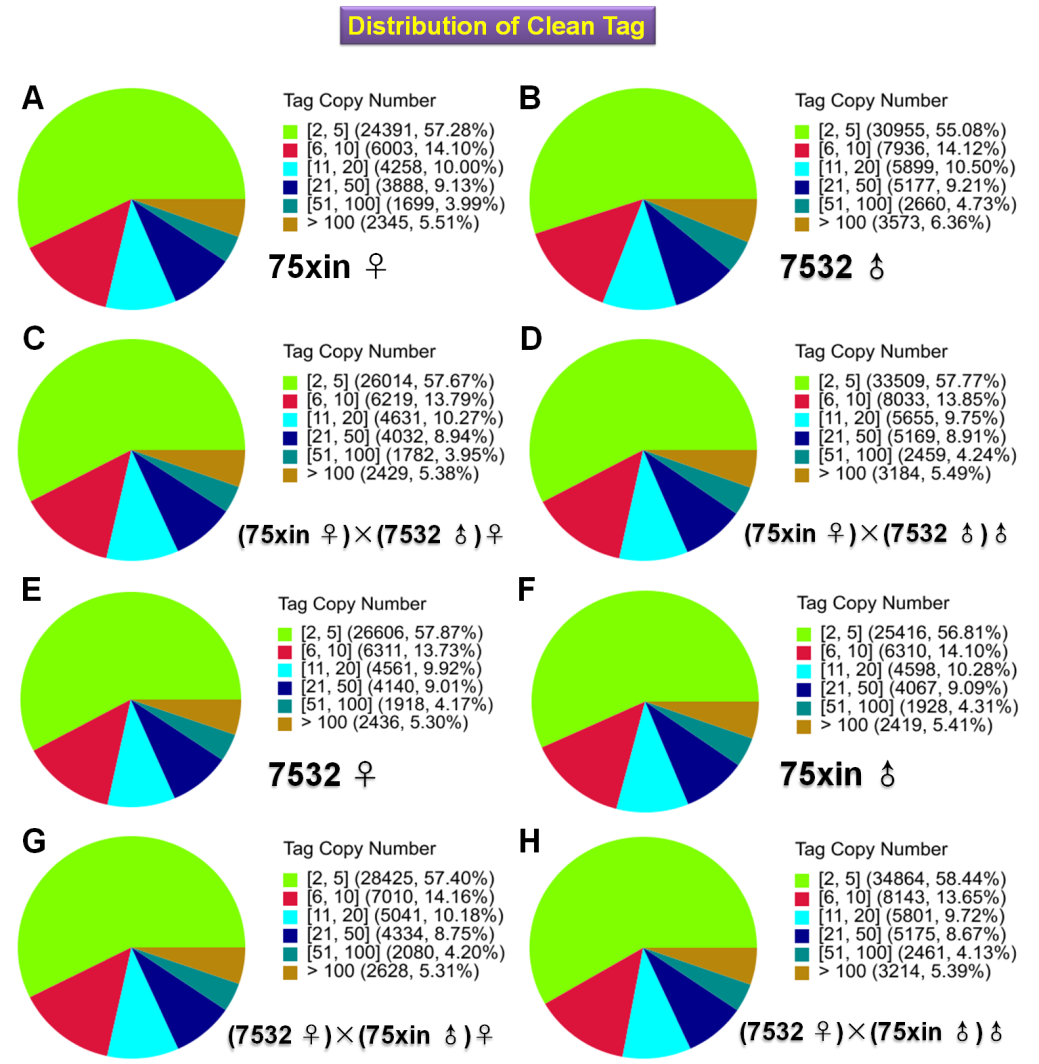


**Figure S3 Distribution of clean tags. Heterogeneity and redundancy were two significant characteristics of mRNA expression.** A small percentageof categories of mRNA had very high abundance, while the majority had a low level of expression. The distribution of clean tag expression was used to evaluate the normality of the data.


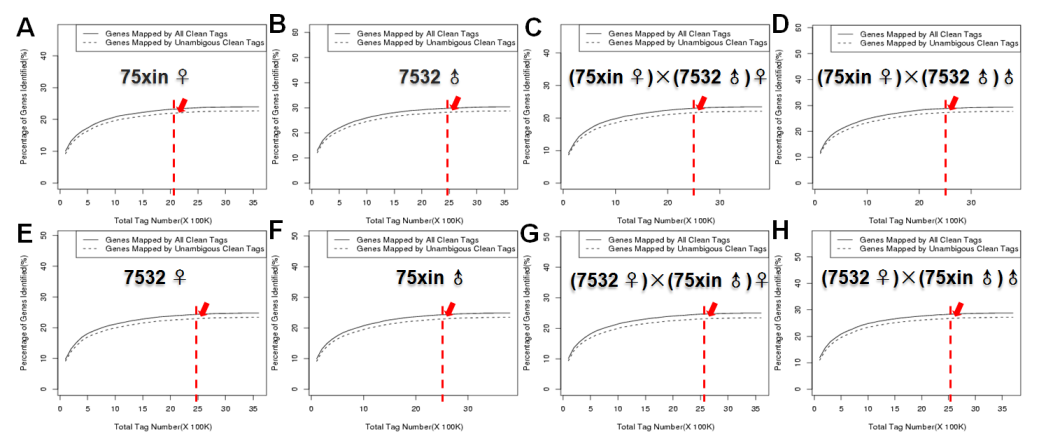


**Figure S4 Saturation analysis of sequencing.** Saturation analysis was performed to check if the number of detected genes increased as sequencing amount (total tag number) increased.


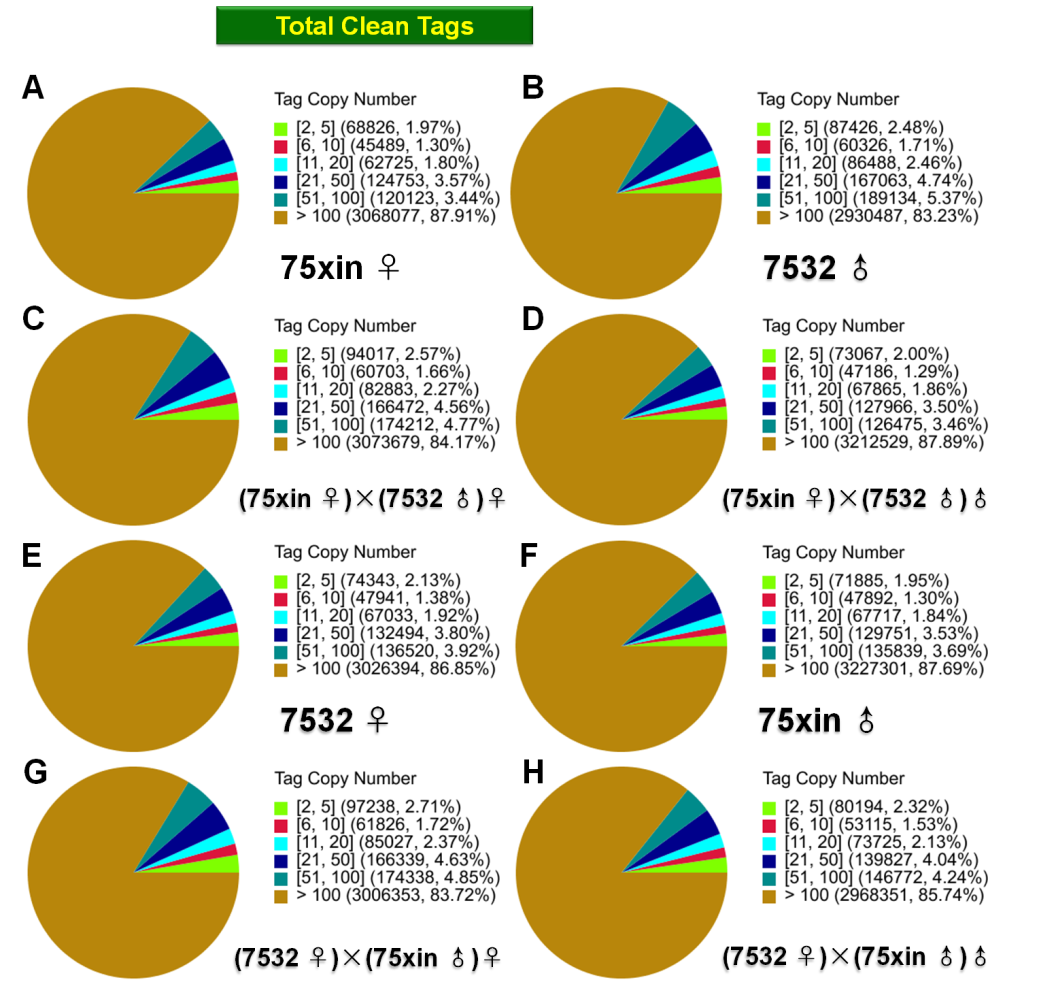


**Figure S5 Distribution of total clean tags.** The numbers in square brackets indicate the range of copy numbers of each tag category. The data in parentheses indicate the percentage of corresponding tags among the total clean tags.


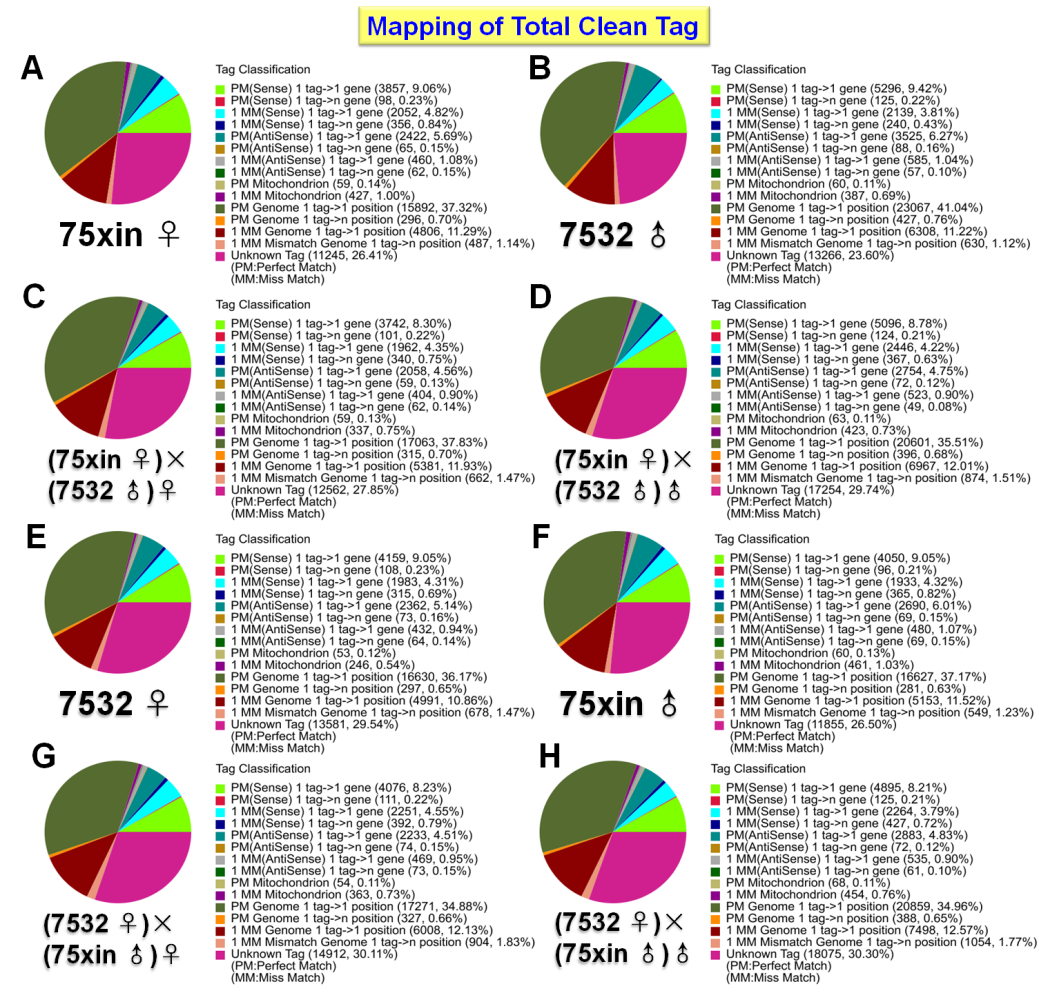


**Figure S6 Mapping of total clean tags.** All clean tags were mapped to reference sequences and only allowed no more than 1 nt mismatch.

**Table S1 Classification Standard of differential gene expression patterns in this study**

| 75xin×”7532” OR  “7532”×75xin Group  Fold | F_1_ Compared with  Female parent(P_1_)  =log2（F_1_-TPM/P_1_-TPM） | F_1_ Compared with  Male parent(P_2_)  =log2（F_1_-TPM/P_2_-TPM） |
| --- | --- | --- |
| No Difference, ND | -2<Fold<2 | -2<Fold<2 |
| Over Parents, OPS | 2≤Fold | 2≤Fold |
| Under Parents, UPS | Fold≤-2 | Fold≤-2 |
| Under Female, UF | Fold≤-2 | -2<Fold<2 |
| Under Male, UM | -2<Fold<2 | Fold≤-2 |
| Over Female, OF | 2≤Fold | -2<Fold<2 |
| Over Male, OM | -2<Fold<2 | 2≤Fold |
| Between Female and Male, FAM | Fold≤-2 | 2≤Fold |
| Between Male and Female, MAF | 2≤Fold | Fold≤-2 |

**Table S2** Primers for qPCR

| Gene | Sense | Anti-sense | Product |
| --- | --- | --- | --- |
| BGIBMGA010975 | CCAATGGAACAGTGGGAGC | GTCATCATCAGGCGTTACAG | 94 |
| BGIBMGA012524 | GCCTCCCTATCAAAAGCAAC | CCCTCACAGAACCCTGGCTGAG | 144 |
| BGIBMGA013545 | GGCTACGACACTTGAGGAATCTG | TGAGGTGGCTTGCCTGATG | 125 |
| BGIBMGA010172 | CTCCTTCTCAACACAGCCCTAT | GCAGTAACATTTCCTCCACTTCTTC | 165 |
| BGIBMGA014427 | TCTTGCCGCTATGGTGATTACT | GCGTGCGTCCTTGAGAGTTC | 128 |
| BGIBMGA007230 | TACCGATTCCCCTACCTC | CTGCTTTCTCCGCCTCCA | 102 |
| BGIBMGA003210 | CGAGCACTCAGCGGGACA | TACACCCACTATCTAACAACCA | 140 |
| BGIBMGA010732 | ACCACTTCAACCAGCCCG | GACACTGTAACGCCGACC | 126 |
| BGIBMGA010722 | ATCGGCTGGGTCGGAAAC | GCTGGCGAGGCACTTGGT | 127 |
| BGIBMGA010275 | GGAAACTGAAACCGAGACT | TCAACAAGCCATCAAACC | 311 |
| BGIBMGA012774 | CCGCAGAAACTAAAACACCCTAAC | GCCGTCTCTCTTTGAACCACAT | 146 |
| BGIBMGA011868 | TCTGGAGTCTGCCTTTAC | GCGTGCTCTGTCTGTGTT | 132 |
| BGIBMGA000066 | GCATCAATCGGATCGCTATG | GGACCTTACGGAATCCATTTG | 147 |

**Table S3 P value of correlation analysis**

| **P value** | **UM** | **OM** | **UF** | **OPS** | **OF** | **UPS** | **MAF** | **FAM** |
| --- | --- | --- | --- | --- | --- | --- | --- | --- |
| **Pupae weight** | 0.068967 | 0.167353 | 0.477 | 0.174811 | 0.474599 | 0.46791 | 0.019403 | 3.39E-05 |
| **Cocoon shell weight** | 0.30629 | 0.143714 | 0.027 | 0.137304 | 0.032773 | 0.025539 | 0.000416 | 6.65E-07 |
| **Whole cocoon weight** | 7.52E-06 | 2.28E-05 | 2E-04 | 2.42E-05 | 0.00013 | 0.00017 | 0.010972 | 0.221616 |
| **Cocoon shell percentage** | 2.27E-06 | 6.87E-06 | 5E-05 | 7.3E-06 | 3.93E-05 | 5.15E-05 | 0.00343 | 0.443258 |
| **Length of fiber** | 1.71E-07 | 5.17E-07 | 4E-06 | 5.48E-07 | 2.95E-06 | 3.87E-06 | 0.000264 | 0.118082 |
| **Diameter of silk fiber** | 4.33E-07 | 1.31E-06 | 9E-06 | 1.39E-06 | 7.49E-06 | 9.83E-06 | 0.000666 | 0.233495 |
